# Individual and Contextual Correlates of Latent Bystander Profiles toward Racist Hate Speech: A Multilevel Person-centered Approach

**DOI:** 10.1007/s10964-024-01968-x

**Published:** 2024-03-18

**Authors:** Sebastian Wachs, Alexander Wettstein, Ludwig Bilz, Dorothy L. Espelage, Michelle F. Wright, Manuel Gámez-Guadix

**Affiliations:** 1 Institute of Education, University of Münster, Münster, Germany; 2 Institute for Research, Development and Evaluation, Bern University of Teacher Education, Bern, Switzerland; 3 Department of Health Sciences, Brandenburg University of Technology Cottbus-Senftenberg, Brandenburg, Germany; 4 School of Education, University of North Carolina at Chapel Hill, Chapel Hill, USA; 5 Department of Psychology, Indiana State University, Terre Haute, USA; 6 Department of Psychology, Autonomous University of Madrid, Madrid, Spain

**Keywords:** Hate speech, Bystanders, School climate, Social skills, Latent profile analysis, Multilevel modelling

## Abstract

Prior research into bystander responses to hate speech has utilized variable-centered analyses — such approaches risk simplifying the complex nature of bystander behaviors. Hence, the present study used a person-centered analysis to investigate latent hate speech bystander profiles. In addition, individual and classroom-level correlates associated with the various profiles were studied. The sample included 3225 students in grades 7–9 (51.7% self-identified as female; 37.2% with immigrant background) from 215 classrooms in Germany and Switzerland. The latent profile analysis revealed that four distinct profiles could be distinguished: *Passive Bystanders* (34.2%), *Defenders* (47.3%), *Revengers* (9.8%), and *Contributors* (8.6%). Multilevel logistic regression models showed common and distinct correlates. For example, students who believed that certain social groups are superior were more likely to be *Revengers* and *Contributors* than *Passive Bystanders*, students who felt more connected with teachers were more likely to be *Defenders*, and students who were more open to diversity were less likely to be *Contributors* than *Passive Bystanders*. Students were less likely *Defenders* and more likely *Revengers* and *Contributors* than *Passive Bystanders* in classrooms with high rates of hate speech perpetration. Further, in classrooms with high hate speech intervention, students were more likely to be *Defenders* and less likely to be *Contributors* than *Passive Bystanders*. In classrooms with stronger cohesion, students were more likely to be *Defenders* and less likely to be *Contributors* than *Passive Bystanders*. In conclusion, the findings add to our understanding of bystander profiles concerning racist hate speech and the relevance of individual and classroom-level factors in explaining various profiles of bystander behavior.

## Introduction

Young people encounter hate speech in multifaceted roles. They find themselves as targets, perpetrators, or, more commonly, as bystanders to hate speech. The bystander’s role is particularly complex, encompassing a spectrum of potential responses ranging from passive inaction to active reinforcement of the perpetrator (Wachs et al., 2024). Despite its prevalence, the intricate spectrum of bystander behavior in the context of hate speech remains underexplored. Investigations into adolescent responses to hate speech have predominantly utilized a variable-centered approach, categorizing individuals into discrete roles based on their responses to specific items or scales. While providing initial insights, this methodology may oversimplify the rich tapestry of bystander responses. A more sophisticated person-centered approach, such as latent profile analysis (LPA; Ferguson et al., 2020), allows the examination of patterns and subgroups of hate speech bystanders across various scales and common responses. Hence, the first objective of this study was to expand the nascent field of hate speech research by employing a person-centered approach to identify distinct profiles of hate speech bystanders among adolescents. In addition, personal factors (e.g., social skills, attitudes toward diversity) but also classroom-level factors (e.g., classroom climate) might play an essential role in understanding students’ responses to hate speech (Ballaschk et al., 2021; Wachs et al., 2022b). Thus, the second objective of this study was to examine, through a socio-ecological lens, how individual and classroom-level factors are related to different bystander profiles.

### Bystander Responses to Hate Speech

Hate speech encompasses various means of communication, such as spoken or written language, graffiti, online commentary, and visual imagery, that intentionally endorses, rationalizes, or spreads animosity and bias against specific social groups and minorities, including LGBTQI+ individuals and people of color, across both digital and physical spaces (Kansok-Dusche et al., 2023). Current research indicates that being a bystander is the most frequent form of involvement in hate speech. In a study among German and Swiss high school students, 67% reported witnessing hate speech in their schools at least once in 12 months, 33% stated they had been targets of hate speech, and 21% reported they had perpetrated hate speech themselves (Castellanos et al., 2023). Bystander responses toward hate speech can vary, but comforting the victim was the most frequently endorsed response when witnessing hate speech in schools, according to recently conducted research (Wachs et al., 2024). This was followed by countering hate speech, ignoring the incident, seeking help at school, helplessness, revenge, and reinforcing. While this research was an essential step in understanding the various responses of students toward hate speech and the frequencies of each response, it did not consider potential subgroups and failed to identify behavior patterns across the six subscales. To fill this gap in research, utilizing person-centered approaches to ascertain distinct profiles of hate speech bystanders across single responses is needed.

In bullying research, bystander behavior has been investigated using person-centered data analyses. For example, in a study examining bystander behaviors in bullying incidents, students were categorized based on their response patterns using latent class analysis on data from over 18,000 high school students (Waasdorp & Bradshaw, 2018). The results revealed that 10% of students exhibited a passive reaction to bullying, 20% reported defending, and 3% acknowledged contributing to the bullying in some way, such as assisting or reinforcing it. Furthermore, they discovered that 65% of the students in their sample did not report any specific response to bullying, indicating limited involvement, while 2% demonstrated an inconsistent response pattern. These findings from bullying research underscore the importance of considering the diverse range of bystander responses in similar contexts, such as hate speech.

### Understanding Hate Speech Bystander Behavior from a Socio-ecological Perspective

The socio-ecological model (Bronfenbrenner, 1979) is a framework to understand human development and behavior by considering the influence of various layers of people’s characteristics, relationships, and social contexts. The central tenet of the socio-ecological model is that multiple levels of influence, including intrapersonal, interpersonal, community, and societal factors, contribute to human behavior.

#### Intrapersonal level

At the individual level, bystanders’ characteristics (e.g., social skills) and attitudes (e.g., social dominance orientation, openness to diversity) may explain their specific responses to hate speech. Social skills (e.g., perspective-taking, prosocial behavior, and assertiveness) might be directly related to bystander behavior because they encompass the social and emotional competencies that enable individuals to act in a caring, empathetic, and socially responsible manner.

Perspective-taking refers to the capacity to comprehend a situation or empathize with other individuals’ thoughts, beliefs, or emotions from a different standpoint (Davis, 1983). It can be assumed that bystanders who are more likely to empathize with targets of hate speech are more likely to defend the targets and less likely to show a passive or aggressive response (e.g., reinforcing or assisting the student who perpetrates hate speech). Prosocial behavior may be linked to bystander behavior because it encompasses actions intended to benefit others, such as helping, sharing, comforting, and expressing concern. When bystanders exhibit prosocial behavior in response to hate speech, they are more likely to intervene in a supportive way toward the target. This can manifest as standing up for the target, offering comfort, or seeking help. Assertiveness is an essential prosocial ability that may empower bystanders to oppose hate speech confidently and respectfully. Those who are assertive can express their disapproval of such conduct calmly and without being aggressive (Kanning, 2003). Today, there is limited empirical evidence on the association between social skills and bystander behavior in the context of hate speech. One study revealed a positive connection to young people’s willingness to counter hate speech (Wachs et al., 2023b). Other research found that empathy was positively related to countering hate speech, searching for help at school, and supporting the target but was negatively associated with reinforcing the perpetrator, revenge, helplessness, and ignoring (Wachs et al., 2024).

Another intrapersonal factor associated with bystander behavior is attitudes, such as openness to diversity and social dominance orientation. Openness to diversity refers to a general attitude of being aware of and accepting the similarities and differences among people (Bayram Özdemir et al., 2021). A meta-analysis revealed that openness to diversity positively relates to tolerance and negatively correlates with prejudices (Ng et al., 2021). These findings align with research that showed that adolescents who harbor negative attitudes and lower tolerance towards immigrants tented to engage in more ethnic harassment and are more accepting of aggressive behavior (Bayram Özdemir et al., 2016; Piumatti & Mosso, 2017).

Additionally, other research demonstrated that youth’s acceptance of diversity was positively associated with their active defending in bullying and negatively related to seeking support from adults, and there was no significant relationship between acceptance of diversity and avoidant bystander responses (Konishi et al., 2021). More recently, a study on hate speech showed that openness to diversity was negatively correlated with hate speech perpetration in schools (Kansok-Dusche et al., 2023). Considering this previous research, one can assume that students with higher levels of openness to diversity are less likely to ignore hate speech or join it and more likely to engage against it when witnessing it.

Social dominance orientation refers to the individual attitude or value system that includes the belief that certain social groups are naturally superior or inferior and that social inequality is justified (Sidanius & Pratto, 1999). Individuals with high social dominance orientation tend to justify and support discriminatory behavior to maintain or strengthen the existing social hierarchy and tend to accept and promote hierarchical structures in society. They believe that some groups are inherently superior and have the right to dominate others. Whether social dominance orientation impacts bystanders’ behavior toward racist hate speech among students has not been investigated. However, previous research revealed that social dominance orientation is positively associated with outgroup prejudice and the acceptance of hate speech (Bilewicz et al., 2017), as well as hate speech perpetration (Castellanos et al., 2023) and bullying perpetration (Volk et al., 2021). Hence, it can be assumed that bystanders high in social dominance orientation are less opposed to hate speech and more likely to participate in hate speech or ignore hate speech incidents when witnessing it, as they believe that some groups are inherently superior and have the right to dominate others. On the other hand, students with low levels of social dominance orientation might be more likely to counter hate speech and support representatives of the targeted group.

#### Interpersonal level

The interpersonal level considers relationships and interactions between individuals, such as the teacher-student relationship. The teacher-student relationship can be defined as the academic, emotional, and interpersonal connection between a student and a teacher (Pianta, 1999). A robust body of research suggests that the complex dynamics of the teacher-student relationship influence the social behaviors exhibited by students, particularly in the context of bystander behavior in bullying situations. More specifically, this research found that a positive teacher-student relationship, characterized by the degree of warmth, closeness, and open communication between the teacher and student, increased the likelihood of a student adopting the role of a prosocial bystander, showing support in favor of the victims. In contrast, a distant or conflictual teacher-student relationship may inadvertently perpetuate a climate of fear or indifference, increasing passive or aggressive bystander behavior (Konishi et al., 2021; Mulvey et al., 2019; Thornberg et al., 2018). Given these findings, it can be assumed that students who experience a positive teacher-student relationship might be less likely to respond passively to hate speech or join in but more likely to support the target and act against hate speech.

#### Contextual factors

In schools across Germany and Switzerland, it is customary for students to remain with the same cohort of peers throughout the school day. As a result, the classroom climate could play a significant contextual role in interpreting the behaviors of bystanders. The classroom climate encompasses various dimensions, including the physical appearance of the classroom, academic monitoring of student progress, and the quality of interpersonal relationships (Loukas, 2007). One crucial element of the social dimension is group cohesion, which reflects students’ collective feelings toward their classmates. In high-group cohesion classrooms, students share common values, support, and care for each other (Leo et al., 2023). As a result, it is likely that in classrooms with solid group cohesion, students are less likely to tolerate hate speech and ignore such incidents and more likely to counter hate speech if students violate social norms of fairness, helping, and mutual respect. Indeed, initial research revealed that in classrooms with higher cohesion, students were more likely to counter hate speech (Wachs et al., 2023b). In addition, other research found that group dynamics within the classroom influence the occurrence of hate speech and partially explained why students join in perpetrating hate speech (Ballaschk et al., 2021; Wachs et al., 2022b).

Despite the classroom cohesion, the occurrence of hate speech in classrooms might play an essential role in understanding bystander behavior. For example, in classrooms with higher hate speech perpetration, students may establish a norm that tolerates or even encourages such behavior, leading to less active intervention or even participation in the hate speech (Ballaschk et al., 2021). Conversely, in classrooms with greater hate speech intervention, students may be more likely to model these responses. Previous bullying research revealed mixed findings in this regard. In research exploring the dynamics of classroom behavior and its impact on bullying, it was discovered that bullying occurrences were less frequent in environments where defending behavior was more observable, while higher instances of bullying correlated with classrooms exhibiting more reinforcing behaviors (Salmivalli et al., 2011). Contrarily, a positive correlation was found between reinforcing behaviors at the classroom level and bullying perpetration, yet no significant relationship was found concerning defending behaviors at the same level (Thornberg & Wänström, 2018). Additionally, no significant association was observed between victimization and bullying at the classroom level with the roles of being a defender or passive bystander (Pozzoli et al., 2012).

## Current Study

The present study is situated within the burgeoning field of hate speech research that seeks to elucidate the nuanced roles of bystanders within schools. Recognizing the multifaceted nature of bystander behavior, this research adopts a person-centered approach, posited to advance the field by capturing the complexity inherent in students’ responses to hate speech incidents. Accordingly, this study’s objectives were twofold. First, the present study seeks to identify latent profiles of adolescent bystanders in response to racist hate speech incidents within school environments. This objective is grounded in the idea that bystander behavior is not monolithic but consists of various response patterns that can be systematically categorized. This study’s second objective is the examination of the correlates of these latent bystander profiles at both the student and classroom levels. Informed by a socio-ecological perspective, the research endeavors to uncover the intrapersonal, interpersonal, and classroom factors associated with various bystander profiles. Given the lack of previous research using latent profile analysis for investigating hate speech bystander behavior and the explorative character of this data analytical approach, no specific hypotheses were formulated.

## Methods

### Participants

The present sample is based on 3225 adolescents (approximately between 12 and 15 years old) from Germany (*n* = 1841; 57.1%) and Switzerland (*n* = 1384; 42.9%). Participants were in grades 7 to 9 (7^th^ grade: 33.2%, *n* = 1070; 8^th^ grade: 35.6%, *n* = 1147; 9th grade: 31.3%, *n* = 1008). In terms of gender, 46.1% (*n* = 1487) self-identified as boys, 51.7% (*n* = 1668) as girls, 2% (*n* = 64) as gender diverse, and 0.2 (*n* = 6) did not indicate their gender. Regarding immigrant background, 40.3% (*n* = 1301) had an immigrant background, and 59.7% (*n* = 1924) did not have an immigrant background. In total, 30.8% (*n* = 994) of students reported living in families of low affluence, 35.8% (*n* = 1155) in families of medium affluence, and 32.4% (*n* = 1046) in families of high affluence. For 0.9% (*n* = 30) of all participants, socioeconomic status (SES) could not be established due to missing values.

### Measures

All measures were presented to the participants in German.

#### Bystander responses to racist hate speech

The Multidimensional Responses to Racist Hate Speech Scale (Wachs et al., 2024) was used to measure young students’ school reactions to hate speech. Participants were presented with a vignette that described a hate speech incident, which reads as follows: “*Please imagine the following situation: At your school, a student makes publicly insulting statements about people of a certain skin color or origin.”* Then the participants were asked: “*What would you do in the situation described, or what have you done if you have experienced such a situation before?*” Following this question, participants were asked to rate 21 items. All items could be answered on a five-point response scale ranging from *strongly disagree* to *strongly agree*. This psychometrically validated instrument includes seven subscales, namely comforting the victim (3 items; McDonald’s ω = 0.84), seeking help at school (3 items; McDonald’s ω = 0.85), countering hate speech (4 items; McDonald’s ω = 0.81), revenge (3 items; McDonald’s ω = 0.84), reinforcing (3 items; McDonald’s ω = 0.65), ignoring (2 items; Spearman-Brown correlation = 0.76), and helplessness (3 items; McDonald’s ω = 0.77).

### Student-Level Measures

#### Social skills

Three subscales measured participants’ self-reported ability to handle social interactions effectively, which was adapted to the school context. The ability to adopt perspectives was measured with a scale of three items (e.g., *I can imagine how schoolmates feel when they were insulted*”; Jurkowski & Hänze, 2014). McDonald’s ω was 0.72. Prosocial behavior was measured with three items (e.g., “*If something bad happens to a classmate, I cheer the person up*”; Jurkowski & Hänze, 2014). McDonald’s ω was 0.81. A scale of five items measured assertiveness, the subjective assessment of being able to influence social situations and their outcomes (e.g., “*When we have a discussion, I can easily get my classmates excited about my suggestions,”* “*If something bothers me in my class, I can do something about it”*; Satow & Schwarzer, 2003). McDonald’s ω was 0.85. Response options for all scales were on a 5-point Likert-type scale ranging from *never* to *always*.

#### Attitudes

Social dominance orientation was measured using a four-item scale (Klocke, 2012). Students rated their level of agreement with four statements that reflected high social dominance orientation (e.g., “It’s *probably ok that certain groups are at the top of society and others at the bottom*”) and four statements that reflected low levels of social dominance orientation (e.g., “*It would be good if all groups were equal*”). Statements that reflected low levels of social dominance orientation were reversed to construct an aggregated score of social dominance orientation. McDonald’s ω was 0.78. Openness to diversity was measured with a scale including two items (e.g., “It’s *ok if schoolmates live a different way of life (e.g., because of their religion) than me,”* Jurkowski & Hänze, 2014). The Spearman-Brown correlation was 0.70. Response options for all scales were on a 5-point Likert-type scale ranging from *never* to *always*.

#### Teacher-student relationship

As one facet of interpersonal relationships in schools, the caring relationship between teachers and students was measured with one scale including six items (e.g., “*At my school, there is a teacher who tells me I do a good job*”; California Healthy Kids Survey, n. d.). Response options were on a 5-point Likert-type scale ranging from *strongly disagree* to *strongly agree*. McDonald’s ω was 0.84.

### Classroom-Level Measures

All the following measures were aggregated at the classroom-level.

#### Frequencies of hate speech

The instrument used to measure hate speech was newly developed for this study. Students were presented with a definition of hate speech in a short video clip. Then, participants received a prompt before the questions, reading as follows:

“*Please only tell us about your experiences that you have had “offline.” That means without the use of digital media. These can be, for example, insulting statements, designations, sayings, threats, insinuations, or graffiti. With hate speech, groups of people (e.g., because of skin color, origin, religion, sexual orientation, or gender) are intentionally insulted or hurt.*”

After this introduction, frequency rates for school hate speech were measured. For the frequency of school hate speech witnessing, participants were asked: “*In the past 12 months, how often have you witnessed hate speech at your school?*” for perpetration, “*…, how often have you perpetrated hate speech at your school?*” for hate speech victimization”*…, how often have you been the target of hate speech at your school?*”, and for hate speech intervention: “*…, how often have you said or done something against hate speech at your school?”* All items were answered on a five-point scale: “*Not at all*” (1), “*1 or 2 times in the last 12 months*”, “*2 or 3 times a month*”, “*about once a week*,” and “*several times a week*” (5).

#### Classroom cohesion

The quality of students’ relationships with their classmates was measured using a three-item scale (e.g., *Most students in my class are friendly and supportive*; Currie et al., 2014). Response options were on a 5-point Likert-type scale ranging from “*absolutely disagree”* to “*absolutely agree*.” McDonald’s ω was 0.82.

### Control Variables

At the student-level, we controlled for gender, immigrant status, and socioeconomic status (SES). Gender was categorized as male or female; gender-diverse participants were excluded due to their small number (*n* = 64). Immigrant status was measured by asking if at least one parent or the participant was born outside Germany or Switzerland (Statistisches Bundesamt Destatis, 2022). Socioeconomic status (SES) was assessed using the Family Affluence Scale (FAS) to categorize low, medium, or high SES based on family assets and holidays (Hartley et al., 2016). At the classroom-level, we controlled for grade, measured directly from student responses regarding their current grade.

### Sampling Technique and Procedure

After obtaining ethical approval for the current study from the University of Potsdam Ethics Committee, an acquisition pool of German sample schools was composed by the federal state of Berlin and Brandenburg, with the type of school (e.g., grammar secondary school [*Gymnasium*] or non-academic-track secondary school [*Realschule*]) being stratified and randomized using the probability-proportional-to-size scheme (Yates & Grundy, 1953). In Switzerland, the acquisition pool of sample schools was designed using a contrastive sampling scheme based on high/low immigrant background and rural/urban geography. From the resulting acquisition pools, 100 schools (Germany: *n* = 76; Switzerland: *n* = 24) were informed via phone calls and e-mails that they had been randomly selected to participate in the study. Acquisition stopped as soon as the sampling plans were fulfilled. In total, 40 schools (Germany: *n* = 18; Switzerland: *n* = 22) agreed to participate. The participation rate at the school level was 40% in the whole sample (Germany: 24%; Switzerland: 92%).

In the present study, 7^th^- to 9^th^-grade students were asked to participate in the survey. In Germany, two randomly selected classes per grade were invited. In Switzerland, all available classes across grades 7 to 9 were invited. In addition, Swiss students in mixed grades were also asked to take part. In total, 264 school classes were invited to participate in the study (Germany: 106; Switzerland: 158). Of these, 236 participated in the study (Germany: *n* = 98; Switzerland: *n* = 138). The response rate at the classroom level was 89% for the whole sample (Germany: 92%; Switzerland: 87%).

Overall, 5836 students (Germany: *n* = 2495; Switzerland: *n* = 3341) were invited to participate in the current study, and 3560 students participated (Germany: *n* = 1841; Switzerland: *n* = 1719). The response rate at the student level was 61% for the whole sample (Germany: 74%; Switzerland: 51%). In total, 335 Swiss students from mixed classrooms in four schools were excluded from the analyses because being in classrooms with mixed grades was confounded with being Swiss.

Data were collected between October 2020 and April 2021 via a tablet-based questionnaire in Germany and online surveys in Switzerland. In Germany, trained research assistants were responsible for collecting the data. Conversely, in Switzerland, schools were provided with an access code to complete the survey. In both countries, data were collected during regular school hours. In the survey, participants first answered questions regarding demographic information, followed by items assessing the frequency of hate speech. Subsequently, the questionnaire explored bystander responses before delving into potential correlates. The rationale for employing a fixed order of measures was chosen to maintain a coherent and logical progression of questions. This approach was intended to facilitate comprehension and engagement among a diverse student sample.

### Data Analyses

#### Missing data analysis

Overall, missing data were between 0.8% (*n* = 30; *hate speech intervention)* and 3.2% (*n* = 113; *assertiveness*). Little’s Missing Completely at Random (MCAR) test revealed that data were not missing at random (χ^2^ = 703.81, *df* = 557; *p* < 0.001). Given the results from Little’s MCAR test that the data are not MCAR, Full Information Maximum Likelihood (FIML) in Mplus was used, that is a suitable and often recommended approach for handling missing data (Enders, 2010).

#### Power analysis

A priori conducted power analysis with G*Power (Faul et al., 2007) revealed that to detect small to medium correlational effect sizes, the present study needed a sample consisting of at least 782 participants (α = 0.05, Power = 0.80). Based on the hierarchical structure of the sample and expected non-response rate, the resulting minimal sample size is *N* = 1944 students in 108 classes at 18 schools. Accordingly, the present sample size was sufficient to investigate the hypotheses.

#### Main analyses

First, LPA was conducted to estimate the likelihood of membership in distinct profiles across seven indicators of bystander responses to racist hate speech (i.e., comforting the victim, seeking help at school, countering hate speech, revenge, reinforcing, ignoring, and helplessness). The standardized mean scale scores of the seven indicators to conduct the LPA were used to ease the interpretation of which indicator values are above or below the sample means. A stepwise approach was applied to determine the number of latent profiles that best characterize the data, starting with an LPA with one profile and successively adding profiles.

To evaluate the model fit, the Akaike information criterion (AIC), the Bayesian information criterion (BIC), and the Sample size-adjusted BIC (SABIC) were used, according to which lower values suggest a better fit (Nylund-Gibson & Choi, 2018; Spurk et al., 2020). Moreover, the adjusted Lo-Mendell-Rubin test (LMR) was utilized. A nonsignificant LMR *p*-value indicates that the solution (k + 1) is not superior compared to the k-profile solution (Nylund-Gibson & Choi, 2018; Spurk et al., 2020). Finally, the Bayes factor (BF) was used as a pairwise comparison of fit between the k-profile and k + 1-profile solutions. The following thresholds were used to interpret the BF: 1 > 3 suggests “weak” support for the model with fewer profiles, 3 > 10 indicates “moderate” support, and > 10 suggests “strong” support (Wagenmakers, 2007). In addition to these model fit criteria, a content-driven aspect to decide on the final number of profiles was applied, which is often recommended practice in LPA (Spurk et al., 2020). As diagnostic criteria, the evaluation of the smallest profile, which should not be smaller than 5% or 50 cases, was used (Spurk et al., 2020). The entropy for which values of < 0.80 indicates a good classification of cases into profiles. The average latent class posterior probability (ALCPP), for which the lowest value should be 0.80 or higher, indicates well-separated classes (Nagin, 2005).

Second, a multilevel multinominal regression analysis (estimator: robust maximum likelihood estimator) was conducted to investigate the associations between student- and classroom-level correlates and the membership on one of the distinct profiles (dependent variable). Multilevel analyses accounted for the nested data structure (students nested within classrooms). First, a null model (Model 0) was run with the classroom as a cluster variable and the multinominal outcome variable obtained through the LPA (k-profile solution). Model 0 was used to get the between-level variances for the random intercepts of the multinominal outcome variable. To calculate the intraclass correlation coefficient (ICC) for each category of the outcome variable, following current research (Burger et al., 2022), the following formula was used (Sommet & Morselli, 2017):

$${\rm{ICC}}=\frac{{var}({u}_{0j})}{{var}\left({u}_{0j}\right)+\frac{{\pi }^{2}}{3}}$$

In this formula, ${u}_{0j}$ represents the random intercept variance. The variance of the logistic distribution was assumed to be $\frac{{\pi }^{2}}{3}\approx 3.29$ because logistic regression models do not provide within-level residuals (Sommet & Morselli, 2017). Then, predictors were entered at the student- and classroom-level (Model 1). At the student-level, the following variables were included: the ability to adopt perspectives, prosocial behavior, openness to diversity, assertiveness, social dominance orientation, and the teacher-student relationship. In addition to this, gender, immigrant background, and SES were entered as control variables. At the classroom-level, hate speech witnessing, perpetration, victimization, and intervention, as well as classroom cohesion were entered while controlling for adolescents’ grade. The LPA and multilevel logistic regression were performed in Mplus Version 8.7 (Muthén & Muthén, 2017).

## Results

### Model Selection and Latent Profiles

Table 1 presents the model fit and diagnostic criteria for the latent profile analysis. As shown in Table 1, the AIC, BIC, aBIC, and the loglikelihood indicated that the five-profiles model had the best fit. Adding one more profile to this solution attained the first nonsignificant LMR-LRT *p*-value (*p* = 0.070), suggesting that the 6-profile model did not show a statistically significant improvement in model fit compared with the 5-profile solution. Comparing the model fits of the first five models revealed that the 5-profile model had the lowest AIC, BIC, and SABIC, indicating the best model fit. However, the five-model solution also had the highest BF (BF = 9.52), suggesting moderate (close to strong) support for the model with one fewer profile, namely the 4-profile solution. When deciding on the final profile solution, it is often recommended to consider both the multiple fit values and content decisions (Spurk et al., 2020). Hence, the 4- and 5-profile solutions were compared to make a content decision. In the 5-profile solution, the additional profile did not add meaningful new insights because the fifth profile showed a pattern similar to the other profile. Therefore, the 4-profile solution was used for subsequent analyses due to parsimony and meaningfulness. Regarding diagnostic criteria, the smallest profile of the 4-profile solution had an acceptable size (8.6%), an adequate entropy (0.81), and the lowest value on the off-diagonal of the average latent class posterior probability was acceptable (0.88).

**Table 1:** Evaluating Latent Hate Speech Bystander Profile Solutions

Model	Model Fit Criteria						Diagnostic Criteria		
	LL	AIC	BIC	SABIC	LMR-LRT *p*-value	BF	Smallest Profile	Entropy	ALCPP
1 Profile	−31613.95	63255.90	63340.88	63296.39	–	–	–	–	–
2 Profiles	−30384.69	60813.38	60946.92	60877.01	< 0.001	3.31	47%	0.70	0.91
3 Profiles	−29576.09	59212.18	59394.28	59298.95	< 0.001	2.17	17%	0.79	0.91
4 Profiles	−29286.98	58649.96	58880.61	58759.87	< 0.001	4.10	9%	0.81	0.88
5 Profiles	−29034.19	58160.39	58439.61	58293.45	< 0.001	9.52	8%	0.77	0.86
6 Profiles	−28807.02	57722.04	58049.82	57878.23	0.070	4.86	3%	0.79	0.84

*N* = 3183. *LL* Loglikelihood, *AIC* Akaike information criterion, *BIC* Bayesian information criterion, *SABIC* Sample-size adjusted BIC, *LMR-LRT* Lo-Mendell-Rubin adjusted likelihood ratio test, *BF* Bayes factor, *ALCPP* Average latent class posterior probability

**Fig. 1:**
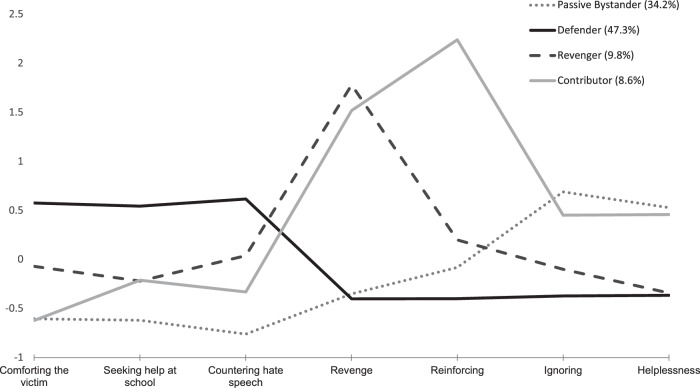
Standardized Means of the Indicators of the Four Latent Hate Speech Bystanders Profiles

Figure 1 shows the z-standardized means of the indicators (below and above the mean) separated by profile membership. The first profile was the second largest, called *Passive Bystander* (34.2%, *n* = 1092). The *Passive Bystander* profile can be characterized by below-average means of comforting the victim, seeking help at school, countering hate speech, below-average means of revenge, reinforcement, and above-average levels of ignoring and helplessness. The second profile was the largest group, called *Defender* (47.3%, *n* = 1515). The *Defender* profile can be characterized by above-average levels in comforting the victim, seeking help at school, and countering hate speech, below-average means in revenge, reinforcin*g*, ignoring, and helplessness. The third profile was the third largest, called the *Revenger* (9.8%, *n* = 314). The profile of *Revenger* can be characterized by below-average means in comforting the victim, seeking help at school, ignoring, and helplessness, close to average means in countering hate speech and reinforcing, and above-average levels of revenge. Finally, the last profile was the smallest, called the *Contributor* (8.6%, *n* = 276). The *Contributors* reported below-average levels of prosocial responses (i.e., comforting the victim, seeking help at school, countering hate speech*)*, above-average levels of antisocial responses (i.e., revenge, reinforcing), and above-average levels of avoidant responses (i.e., ignoring, helplessness*)*.

### Correlates of Hate Speech Bystander Profiles

Table 2 presents the findings of the multilevel multinominal regression analysis. The *Passive Bystander* profile was used as the reference category. Selecting the *Passive Bystander* profile as the reference category was grounded in the research and practical interests outlined in the literature review. In addition, the present study aimed to investigate the factors contributing to an individual’s transition from passive observation to active involvement, whether positively (as *Defender*) or negatively (as *Revenger* or *Contributor*), in the context under study. This choice was informed by the existing body of literature that suggests a critical need to understand what motivates bystanders to move beyond passivity in situations where their action or inaction can have significant outcomes.

**Table 2:** Results of a Multilevel Multinomial Logistic Regression Analysis Predicting Latent Hate Speech Bystander Profiles

Variable	Defender vs. Passive Bystander				Revenger vs. Passive Bystander				Contributor vs. Passive Bystander			
	Est.	SE	*p*	OR	Est.	SE	*p*	OR	Est.	SE	*p*	OR
Model 0												
Intercept	0.25	0.06	<0.001		−1.27	0.08	<0.001		−1.64	0.12	<0.001	
Variance	0.43	0.07	<0.001		0.09	0.10	0.371		0.63	0.18	<0.001	
Intraclass correlation (ICC)	0.11				0.03				0.16			
Model 1												
Student-level												
Intrapersonal factors												
Ability to adopt perspectives	**0.34**	**0.06**	**<0.001**	**1.41**	**−0.20**	**0.09**	**0.022**	**0.82**	−0.05	0.13	0.690	0.95
Prosocial behavior	**0.39**	**0.07**	**<0.001**	**1.48**	0.15	0.12	0.210	1.16	−0.09	0.12	0.440	0.91
Assertiveness	**0.19**	**0.06**	**<0.001**	**1.21**	**0.55**	**0.10**	**<0.001**	**1.74**	**0.66**	**0.11**	**<0.001**	**1.94**
Openness to diversity	0.09	0.06	0.177	1.09	−0.12	0.10	0.241	0.89	−**0.36**	**0.10**	**<0.001**	**0.70**
Social dominance orientation	0.06	0.05	0.237	1.06	**0.21**	**0.07**	**0.003**	**1.24**	**0.27**	**0.09**	**0.002**	**1.31**
Interpersonal factor												
Teacher-student relationship	**0.21**	**0.05**	**<0.001**	**1.23**	0.08	0.09	0.389	1.08	0.01	0.07	0.987	1.00
Control variables												
Gender ^girls^	**0.65**	**0.10**	**<0.001**	**1.91**	−**0.88**	**0.18**	**<0.001**	**0.42**	−**0.71**	**0.18**	**<0.001**	**0.48**
Immigrant Background ^yes^	−0.18	0.10	0.089	0.84	**0.59**	**0.14**	**<0.001**	**1.81**	**0.47**	**0.18**	**0.008**	**1.59**
SES	0.06	0.07	0.407	1.06	0.06	0.09	0.509	1.06	0.03	0.10	0.785	1.03
Classroom-level												
HS witnessing	−**0.52**	**0.14**	**<0.001**	–	−0.39	0.22	0.074	–	−0.31	0.21	0.151	–
HS perpetration	−**0.79**	**0.25**	**0.002**	–	**0.64**	**0.24**	**0.007**	–	**1.23**	**0.34**	**<0.001**	–
HS victimization	−0.23	0.27	0.395	–	−0.14	0.29	0.630	–	−0.43	0.33	0.193	–
HS intervention	**0.86**	**0.24**	**<0.001**	–	0.59	0.34	0.084	–	−**0.80**	**0.23**	**<0.001**	–
Classroom cohesion	**0.39**	**0.18**	**0.031**	–	0.13	0.24	0.591	–	−**1.19**	**0.26**	**<0.001**	–
Grade	−0.22	0.08	0.004	–	0.06	0.10	0.559	–	−0.17	0.12	0.154	–

*Note*. *N*_students_ = 2988; *N*_classrooms_ = 214, HS = hate speech; OR = odds ratio; Est. = raw estimates. Model 0: AIC = 7260.76; BIC = 7297.18, Loglikelihood H_0_ = −3624.38, H_0_ Scaling correction factor for robust maximum likelihood estimation (MLR) = 0.980. Model 1: AIC = 7377.16; BIC = 7749.31, Loglikelihood H_0_ = −3626.58, H_0_ Scaling correction factor for robust maximum likelihood estimation (MLR) = 1.792. At the classroom-level, there is a random intercept for the multinominal dependent variable (latent random intercept); estimates represent linear regression slopes. Bold means significant effect

The analysis of the baseline model (Model 0) showed that for being a *Defender*, the ICC was 0.11; for being a *Revenger*, the ICC was 0.03; and for being a *Contributor*, the ICC was 0.16, indicating that 11, 3, and 16% of the variance in the likelihood of adopting a specific profile in comparison to being a *Passive Bystander* could be explained due to class differences. As shown in Model 1 (see Table 2), several significant student- and classroom-level correlates emerged.

#### Defenders

At the student-level, higher levels of ability to adopt perspectives, prosocial behavior, assertiveness, and teacher-student relationship were positively associated with being a *Defender*. At the classroom-level, higher hate speech witnessing and hate speech perpetration were negatively related to being a *Defender*. In addition, hate speech intervention and classroom cohesion were positively linked to being a *Defender*. In other words, defending was less frequent in classrooms where more hate speech was observed and perpetrated by students. Whereas in classrooms where more students intervened, and cohesion was stronger, defending was more frequent.

#### Revengers

At the student-level, higher levels of ability to adopt perspectives were negatively related, and higher levels of assertiveness and social dominance orientation were positively associated with being a *Revenger*. At the classroom-level, higher hate speech perpetration frequencies were positively linked to being a *Revenger*. In other words, more students were *Revengers* in classrooms where more hate speech was perpetrated.

#### Contributors

At the student-level, assertiveness and social dominance orientation were positively associated with being a *Contributor*. Openness to diversity was negatively related to being a *Contributor*. At the classroom-level, higher frequencies of hate speech perpetration were positively associated with being a *Contributor*. In contrast, higher levels of hate speech intervention and classroom cohesion at the classroom-level were negatively linked to being a *Contributor*. In other words, contributing was more frequent in classrooms where students perpetrated more hate speech. In classrooms where more students intervened, and cohesion was stronger, contributing was less frequent.

## Discussion

Many students are confronted with hate speech in schools. Most students are witnesses of hate speech and are not directly involved as perpetrators or targets themselves (Castellanos et al., 2023). To date, no research has investigated bystander responses to hate speech through more advanced person-centered approaches that allow identifying subgroups of adolescent bystanders with similar response patterns. Therefore, the first objective of this study was to use latent profile analysis to identify different bystander profiles among a large sample of adolescents. Understanding the individual and classroom-level characteristics of these varied hate speech bystander profiles is also essential. Toward that end, a secondary objective of the present study was to use multilevel logistic regression modeling to identify individual and classroom-level correlates of latent bystander profiles.

### Hate Speech Bystander Profiles

Regarding the first research objective, the analyses delineated four distinct profiles of bystander behaviors responding to hate speech, each with unique behavioral patterns. These profiles — *Passive Bystander*, *Defender*, *Revenger*, and *Contributor* — offer a comprehensive view of how adolescents may react to racist hate speech incidents and provide a framework for understanding the complexities of bystander behavior.

#### Passive bystanders

The *Passive Bystander* profile represented just over a third of the sample. The above-average levels of ignoring the situation and feelings of helplessness are particularly concerning. This indicates that *Passive Bystanders* may recognize the harm but feel ill-equipped to respond or believe their response would be ineffective. This sense of helplessness can be a significant barrier to intervention (Wachs et al., 2023a). While not directly harmful, this neutrality does nothing to challenge the status quo. It can be seen as allowing the perpetuation of a culture of silence around hate speech, where such behavior is not openly challenged.

The below-average levels of countering hate speech, comforting the victim, and seeking help suggest a reluctance to get involved, a tendency to avoid confrontation or a possible lack of awareness about how to respond to such incidents effectively. This could be due to various factors, including fear of retaliation, a sense of futility, a lack of identification with the target or the social group represented by the target, or a lack of confidence in their ability to make a difference or a desire to not draw attention to themselves (Ballaschk et al., 2021; Krause et al., 2021). Interestingly, *Passive Bystanders* also show below-average levels of revenge and reinforcement of the hate speech, which suggests that while they do not contribute to the escalation of the situation, they also do not actively take steps to prevent or diminish it. Their below-average engagement in pro-social responses, coupled with higher levels of avoidant responses, suggests a profile that is either indifferent, lacks the confidence to intervene, or possibly feels disempowered to act. This aligns with previous bystander research (Latané & Darley, 1970).

#### Defenders

The largest group identified was the *Defenders*. The above-average levels of comforting the victim and seeking help from school authorities support the idea that *Defenders* are not only empathetic (Wachs et al., 2023a) but also trust in the school’s support systems to address such incidents. Their willingness to counter hate speech actively demonstrates a sense of moral courage and a commitment to upholding a respectful community ethos. This suggests that *Defenders* feel responsible for intervening. These findings may be explained by previous research that showed that defending bystander responses is positively related to empathy and negatively related to moral disengagement (Wachs et al., 2024). The below-average tendencies in revenge and reinforcing hate speech suggest that *Defenders* are likely to favor constructive responses over those that could exacerbate the situation and understand that such actions could lead to further harm and a cycle of retaliation.

Similarly, their lower likelihood of reinforcing the behavior of the hate speech perpetrator through encouragement or participation suggests a strong stance against hate speech. The lower levels of ignoring the situation and feelings of helplessness among *Defenders* are particularly noteworthy. These findings align with previous research that *Defenders* feel capable and equipped to act, which may result from their characteristics, such as self-efficacy and resilience (Wachs et al., 2023a, b).

#### Revengers

The most defining characteristic of the *Revenger* profile is the above-average levels of revenge. This suggests that individuals in this category may be inclined to take matters into their own hands, responding to hate speech with their form of justice, which could potentially escalate the situation. This retaliatory approach may be driven by anger, a desire for retribution, or social norms within the classroom (Ballaschk et al., 2021; Wachs et al., 2022b). Although not a large group, *Revengers* presents a unique challenge. The identification of the *Revenger* profile within the context of bystander responses to hate speech reveals a complex interplay of behaviors that are both reactive and, potentially, retaliatory. The *Revengers* are notable for their below-average engagement in supportive actions for the victim and in seeking help from school authorities, which are critical components of a constructive bystander response. The lack of prosocial engagement suggests that *Revengers* may not prioritize or value these responses or feel that such actions are ineffective. This could be due to a belief that the formal mechanisms are insufficient to address the issue, or it could stem from a lack of faith in the system’s ability to deliver justice or protection for the target (Ballaschk et al., 2021). Interestingly, *Revengers* are described as having close to average means in countering hate speech. This indicates a nuanced stance where *Revengers* may occasionally speak out against hate speech but are not consistently engaging in behaviors that would actively discourage it. This duality could reflect an internal conflict or a lack of a clear strategy for responding to hate speech incidents.

#### Contributors

The findings regarding the *Contributor* profile, the smallest group, are particularly concerning. The below-average levels of prosocial responses indicate a lack of engagement in supportive actions that could mitigate the harm done to the targets. This is problematic because it suggests a deficit in either the willingness or the ability to engage in behaviors that are generally considered constructive and supportive in the context of hate speech. The above-average levels of antisocial responses, including revenge and reinforcing behaviors, indicate a more troubling aspect of the *Contributor* profile. These students do not merely stand by; they may participate in or encourage the continuation of hate speech. This suggests that *Contributors* may hold beliefs or attitudes that align with the hate speech perpetrator, or they may gain some social or psychological benefit from their involvement in these negative behaviors. These findings are consistent with research that engagement in hate speech is often motivated by achieving status enhancement, exhilaration, or group conformity (Ballaschk et al., 2021; Wachs et al., 2022a). The above-average levels of avoidant responses, such as ignoring the situation or feeling helpless, suggest a complex interaction with the hate speech incidents. While *Contributors* may seem to be actively supporting hate speech through reinforcement, their avoidance behaviors imply a possible internal conflict or a desire to disengage from the direct consequences of their actions. This idea is supported by research that found that moral disengagement positively correlated with bystander responses such as reinforcing, ignoring, or helplessness (Wachs et al., 2024). This avoidance could also reflect a broader social influence, where the norms of the peer group or community may discourage active intervention or may even stigmatize those who stand against hate speech (Ballaschk et al., 2021; Wachs et al., 2023a).

### Individual and Classroom-level Correlates of Hate Speech Bystander Profiles

Regarding the second objective, several individual and classroom-level correlates were found. Since the *Passive Bystander* profile was used as a reference category, the identified correlates indicate an individual’s likelihood to adopt a role other than a passive bystander — as a *Defender*, someone seeking *Revenge*, or a *Contributor* to the situation.

#### Defenders

The current study’s identification of *Defenders* aligns with prior variable-centered research that has consistently highlighted the role of individual traits such as empathy, prosocial behavior, and assertiveness in predicting defending behavior (Wachs et al., 2024; Wachs et al., 2023b). The positive influence of a strong teacher-student relationship on the likelihood of a student becoming a *Defender* also resonates with past findings that supportive adult relationships are crucial to fostering student resilience and bystander intervention in bullying (Konishi et al., 2021; Mulvey et al., 2019; Thornberg et al., 2018). However, the current study extends these findings by showing this association for defending behavior in comparison to passive bystanding in hate speech. In addition, classrooms with high instances of hate speech witnessing and perpetration seem to inhibit the *Defender* role compared to being *Passive Bystanders*. This could be due to a normalization of hate speech, where it becomes an accepted part of the classroom culture, thus discouraging students from taking a stand against it (Ballaschk et al., 2021). Conversely, classrooms with higher levels of intervention and cohesion see an increase in the defender role compared to the passive bystander role. This supports previous research (Wachs et al., 2023a) and indicates that proactive responses to hate speech and a united classroom environment can foster a sense of responsibility and empowerment among students to act in defense of those targeted by hate speech while reducing passive bystanding.

#### Revengers

The association between assertiveness, social dominance orientation, and being a *Revenger* offers a new perspective. While previous research has focused on the role of empathy and moral disengagement in retaliatory behavior (Wachs et al., 2024), the current study suggests that assertiveness and a desire for social dominance can also contribute to revenge-oriented responses compared to passive bystanding in hate speech. This is somewhat supported by research where exercising Power within classrooms is identified as one major driver in the perpetration of hate speech (Wachs et al., 2022a). These findings also expand the little research on the relation between social dominance orientation and hate speech perpetration among young people (Castellanos et al., 2023). The positive association between hate speech perpetration at the classroom level and the *Revenger* profile suggests that environments with frequent hate speech may foster a retaliatory mindset while reducing passive bystanding. This could be a manifestation of a hostile climate where students feel that revenge is a justified or necessary response to hate speech to reduce one’s own vulnerability to become the next target.

#### Contributors

The *Contributor* profile, as identified in the current study, is particularly novel in its emphasis on the negative and unique association with openness to diversity. The current findings extend research revealing a negative link between openness to diversity and the perpetration of hate speech (Kansok-Dusche et al., 2023). Aligning to this, research from related fields indicated that openness to diversity is positively related to tolerance, negatively correlated with prejudices, ethnic harassment, and aggressive behavior (Bayram Özdemir et al., 2016; Ng et al., 2021; Piumatti & Mosso, 2017). In addition, the current study’s findings extend previous knowledge by showing that assertiveness and social dominance orientation are positively associated with being a *Contributor*, adding to prior research on perpetration (Castellanos et al., 2023).

Classrooms with higher rates of hate speech perpetration also have more *Contributors* than *Passive Bystanders*, indicating that such behaviors may be contagious or that students may feel peer pressure to join in (Ballaschk et al., 2021; Wachs et al., 2022b). The negative relationship between hate speech intervention and cohesion with the *Contributor* profile in comparison to the *Passive Bystander* profile suggests that if classrooms collectively counter hate speech, it discourages others from contributing to it. This could be due to a stronger sense of pro-social norms against hate speech and a supportive environment that dissuades negative behaviors (Wachs et al., 2023a).

### Limitations and Outlook on Future Research

Although the present study has many strengths, several limitations must be mentioned. First, the cross-sectional nature of the data allowed the present study to take a static view of latent hate speech bystander profiles. Longitudinal research is needed to understand the development of certain profile memberships, the stability, and potential transitions from one profile to another. Second, only a very few classroom-level correlates were considered. In addition, the items for measuring hate speech frequency referred to the school, not the classroom environment. This might explain why the findings showed relatively few significant associations between the variables measuring hate speech frequencies and latent hate speech bystander profiles. Follow-up research should consider more aspects of the classroom environment and use hate speech frequency items referring to the classroom context. Third, the present study investigated only bystander profiles concerning racist hate speech. Follow-up research should compare the findings to other social groups (e.g., homophobic hate speech) to understand differences and similarities in young people’s bystander profiles. Finally, while most of the subscales of the Multidimensional Responses to Racist Hate Speech Scale showed adequate reliabilities, the McDonald’s ω for the reinforcing subscale was slightly below the recommended cut-off of 0.70. Follow-up research is needed to understand how this instrument can be improved to measure reinforcing in a more reliable way.

### Practical Implications

The findings of this study have important implications for educational practices and policies aimed at fostering positive student interactions and mitigating negative bystander behaviors, such as revenging or contributing and encouraging positive bystander behavior, such as defending within the classroom setting.

#### Implications for passive bystanders

The presence of *Passive Bystanders* in significant numbers can create an environment where hate speech goes unchallenged and becomes normalized. This underscores the importance of educational programs that not only address the perpetrators of hate speech but also empower bystanders to take constructive action. Schools can play a critical role in changing this dynamic by creating a culture where all community members feel responsible for maintaining a respectful and safe environment. This could involve training students in bystander intervention strategies, building a strong sense of community where students feel supported in action, and ensuring that there are explicit, safe, and effective channels for reporting and addressing hate speech. The *Passive Bystander* should not be overlooked in the fight against hate speech. By understanding the reasons behind their inaction, interventions can be tailored to encourage more active and supportive bystander behaviors, thereby reducing the overall tolerance for hate speech within the school community.

#### Implications for defenders

The positive association between student-level factors such as the ability to adopt perspectives, prosocial behavior, assertiveness, and a strong teacher-student relationship with the likelihood of being a *Defender* suggests that educational interventions should focus on enhancing these qualities. Programs that promote perspective-taking, encourage prosocial behavior, and train students in assertiveness could be integral in cultivating *Defenders* within the student body. Moreover, the critical role of the teacher-student relationship implies that teacher training should emphasize relationship-building skills to create a supportive and responsive classroom environment. Furthermore, the results highlight the pivotal role of the teacher-student relationship in promoting defensive actions against hate speech. The findings indicate that teacher engagement is not just beneficial for academic outcomes but is also crucial for fostering a supportive and active bystander culture within schools. At the classroom-level, the negative relation of witnessing hate speech and perpetrating hate speech to being a *Defender* indicates a need for a proactive approach to creating a classroom climate that does not tolerate hate speech. Conversely, the positive link between hate speech intervention and classroom cohesion with being a *Defender* suggests that fostering a sense of community and collective responsibility can encourage students to stand up for one another. The findings also highlight the crucial role of peer models, indicating that students are likely to mirror prosocial behaviors within classrooms.

#### Implications for revengers

The findings regarding *Revengers* highlight the complexity of addressing aggressive responses to hate speech. The negative association between the ability to adopt perspectives and being a *Revenger* suggests that interventions that enhance empathy and perspective-taking may reduce vengeful responses. However, the positive association with assertiveness indicates that these interventions must be carefully designed to channel assertiveness into positive actions rather than aggressive retaliation. The findings also showed that individual beliefs about social hierarchy influence whether students become *Revengers* rather than *Passive Bystanders*. This underscores the importance of addressing underlying social and moral convictions in educational interventions. The classroom-level association between higher hate speech perpetration and being a *Revenger* emphasizes the urgency of educational policies to address the root causes of hate speech. Addressing the needs and behaviors of *Revengers* requires a multifaceted approach. Interventions might include teaching conflict resolution and anger management skills, fostering the ability to adopt perspectives, and creating opportunities for restorative justice that allow for the acknowledgment of harm and active participation in the resolution process. Additionally, reinforcing the effectiveness and availability of supportive measures for dealing with hate speech, such as counseling and reporting mechanisms, could help redirect the Revengers‘ inclination for direct action into more positive channels. Strategies that can be employed to guide students towards more constructive responses might be teaching students how to negotiate, mediate, and resolve disputes peacefully or including role-plays in different scenarios where students can learn effective ways to practice these skills.

#### Implications for contributors

For *Contributors*, the positive association with assertiveness and social dominance orientation and the negative association with openness to diversity suggest that educational strategies should promote inclusivity and respect for diversity while recognizing the complex interplay between assertiveness and classroom behavior. Programs that teach assertive communication that respects diversity and fosters inclusivity could be beneficial. At the classroom-level, the fact that higher frequencies of hate speech perpetration are associated with being a *Contributor*, while higher levels of hate speech intervention and classroom cohesion are negatively associated, suggests that interventions need to target the classroom climate as a whole. Efforts to increase classroom cohesion and collective hate speech intervention could reduce the instances of contributing to hate speech. Adopting a zero-tolerance policy towards hate speech and implementing comprehensive educational programs that address such behavior may be effective strategies.

## Conclusion

Research on bystanders toward hate speech used variable-centered approaches that may overlook the existence of complex patterns of bystander responses. To this end, this study investigated the profiles of adolescent bystanders to racist hate speech, utilizing latent profile analyses. The findings shed light on the nuanced roles that students take on when witnessing hate speech. By identifying four distinct bystander profiles — *Passive Bystanders*, *Defenders*, *Revengers*, and *Contributors* — this research contributes to a more granular understanding of bystander behavior in the context of hate speech. The research findings underscore the need for nuanced intervention strategies tailored to the different profiles. While *Passive Bystanders* may require education and empowerment to become more active*, Defenders* may benefit from continued support and reinforcement of their actions. *Revengers* may need guidance to redirect their assertive tendencies into positive activities, and *Contributors* may require a more fundamental change in attitudes and beliefs about diversity and inclusion. In addition, these findings underscore the need for multifaceted educational interventions that operate at both the student and classroom levels. Such interventions should promote perspective-taking, prosocial behavior, assertiveness, and a strong sense of community while also directly addressing the issues of hate speech and social dominance orientation. By doing so, schools can create environments that discourage hate speech and actively encourage students to support and protect one another.
